# Genetic diversity, distribution, and evolution of chicken anemia virus: A comparative genomic and phylogenetic analysis

**DOI:** 10.3389/fmicb.2023.1145225

**Published:** 2023-03-09

**Authors:** Pir Tariq Shah, Amina Nawal Bahoussi, Xiaogang Cui, Shaista Shabir, Changxin Wu, Li Xing

**Affiliations:** ^1^Institutes of Biomedical Sciences, Shanxi University, Taiyuan, Shanxi, China; ^2^Shanxi Provincial Key Laboratory of Medical Molecular Cell Biology, Shanxi University, Taiyuan, China; ^3^Shanxi Provincial Key Laboratory for Prevention and Treatment of Major Infectious Diseases, Taiyuan, China; ^4^The Key Laboratory of Chemical Biology and Molecular Engineering of Ministry of Education, Shanxi University, Taiyuan, China

**Keywords:** chicken anemia virus, phylogenetics, diversity, distribution, recombination

## Abstract

Chicken infectious anemia (CIA) is an immunosuppressive poultry disease that causes aplastic anemia, immunosuppression, growth retardation and lymphoid tissue atrophy in young chickens and is responsible for huge economic losses to the poultry industry worldwide. The disease is caused by the chicken anemia virus (CAV), which belongs to the genus *Gyrovirus*, family *Anelloviridae*. Herein, we analyzed the full-length genomes of 243 available CAV strains isolated during 1991–2020 and classified them into two major clades, GI and GII, divided into three and four sub-clades, GI a-c, and GII a-d, respectively. Moreover, the phylogeographic analysis revealed that the CAVs spread from Japan to China, China to Egypt and subsequently to other countries, following multiple mutational steps. In addition, we identified eleven recombination events within the coding and non-coding regions of CAV genomes, where the strains isolated in China were the most active and involved in ten of these events. Furthermore, the amino acids variability analysis indicated that the variability coefficient exceeded the estimation limit of 1.00 in VP1, VP2, and VP3 proteins coding regions, demonstrating substantial amino acid drift with the rise of new strains. The current study offers robust insights into the phylogenetic, phylogeographic and genetic diversity characteristics of CAV genomes that may provide valuable data to map the evolutionary history and facilitate preventive measures of CAVs.

## Introduction

Chicken infectious anemia (CIA), caused by the chicken anemia virus (CAV), is an immunosuppressive poultry disease that typically causes aplastic anemia, immunosuppression, growth retardation and lymphoid tissue atrophy (Pope, 1991; Schat, 2009; Liu et al., 2022), causing huge economic losses to the poultry industry all around the world (Schat, 2009; Fatoba and Adeleke, 2019). In adult chickens, CAV causes a mild subclinical infection, however, the infected chickens are usually manifested immunosuppression and become more sensitive to secondary infections with bacterial, viral or fungal pathogens (Adair and Immunology, 2000). It also causes a sub-optimal response to the vaccinations, making it difficult to control (Techera et al., 2021). Though chicken is the main host of CAV and all kinds of chicken breeds are susceptible to this virus, several reports support the presence of CAV in the feces of other birds and some animals, e.g., mice and dogs (Rijsewijk et al., 2011; Chu et al., 2012; Zhang et al., 2014; Fang et al., 2017; Li et al., 2017; Liu et al., 2022). CAV strains generally have a 30% mortality (Li et al., 2021), which may reach 60%, particularly in complicated cases (Gelderblom et al., 1989). CAV mainly infects 10–14 days-old chickens, leading to severe anemia, yellow bone marrow, aplasia of the bone marrow and atrophy of the lymphoid organ by damaging erythroblastoid cells, resulting in depletion of thymocytes, which makes the chickens immunodeficient (Jeurissen et al., 1992; McIlroy et al., 1992; Adair and Immunology, 2000). Another cause of high mortality in chickens is the atrophy of the thymus and bone marrow, which also causes immunosuppression and weight loss in 2–4 weeks-old chicks. Therefore, CAV is considered an important viral agent of avian species worldwide (Kabir et al., 2021).

CAV is a non-enveloped, icosahedral, and single-stranded DNA virus with 23–25 nm in diameter that belongs to the genus *Gyrovirus* of the *Anelloviridae* family (Rosario et al., 2017; Di Francesco et al., 2022). The virus genome is about 2.2–2.3 kb that contains three overlapping Open Reading Frames (ORFs) that encode 51.6 kDa VP1 capsid protein, 24 kDa VP2 associated-protein and 13.6 kDa VP3 apoptin protein (Rosenberger and Cloud, 1998; Lacorte et al., 2007), respectively. VP1 is the major structural protein that contains ample antigenic epitopes and plays a vital role in the growth and transmission of CAV (Renshaw et al., 1996). VP2 is a non-structural protein with phosphatase activity that plays a key role in the virus’s assembly during the infectious cycle. VP2 is also a scaffold protein, which assists in folding the VP1 protein during viral particle assembly. Furthermore, the VP1/VP2 co-expression can stimulate the antibody neutralizing in the host cell (Koch et al., 1995; Peters et al., 2002). VP3 (apoptin protein) is the main virulence factor of CAV, which induces severe lympho-atrophy and anemia in infected chickens, and can trigger apoptosis independent of p53 activation in the host cell and many tumor cell lines (Jeurissen et al., 1992; Zhuang et al., 1995). VP1 and VP2 proteins are the prime targets in designing vaccines to induce neutralizing antibodies (Moeini et al., 2011a).

CAV transmission occurs through horizontal and vertical routes (Miller and Schat, 2004; Gimeno, 2013). Horizontal transmission occurs *via* feathers, feces, oral contamination, and from chicken to chicken, while vertical transmission occurs through breeders to their progeny (McNulty et al., 1991; Davidson et al., 2008). CAV can persist in highly acidic environments reaching up to pH 3, and is resistant to harsh chemicals such as chloroform and acetone, commonly used for disinfection (Goryo et al., 1985). CAV is quite stable at high temperatures, surviving at 80°C for 30 min and inactivating completely at 100°C for 10 min (Urlings et al., 1993). The CAV ubiquity is due to all these characteristics (Todd et al., 1990). Commercial vaccines are currently available in the form of live attenuated type (Batheja et al., 2021), which are effective in overcoming the infection; however, they pose the risk of horizontal and vertical transmission to other chicks (Zeng et al., 2021). The commercially accessible vaccines include the CAV vaccine developed based on non-pathogenic CAV grown in chicken embryos (Vielitz and Landgraf, 1988) and an attenuated live virus strain (Steenhuisen et al., 1994). These vaccines, however, cannot be used on chickens in the laying stage or within 21 days after slaughter. Furthermore, if a live vaccine is not properly attenuated, it can cause clinical disease, and the dissemination of modified viruses to young chicks can also cause disease (Moeini et al., 2011b). Continuous reports of CAV outbreaks due to vaccination failure have resulted in the development of a plethora of contemporary vaccines with possible protection (Batheja et al., 2021).

The first case of CAV was reported in 1979 in Japan, isolated from commercially produced chickens (Yuasa et al., 1979). Since then, the virus has been detected by isolation or serological analysis in many other countries in both laying and broiler chickens (Bulow et al., 1997) and has become a global epidemic, which is being reported in most of the poultry-breeding countries, including Egypt, Italy, and Argentina et al. (Rosenberger and Cloud, 1989; Craig et al., 2009; Abdel-Mawgod et al., 2018; Quaglia et al., 2021; Techera et al., 2021). In China, CAV was first reported in 1996 (Zhou et al., 1997) and then detected subsequently in a chicken flock in Shandong, Guangdong, Jiangsu and many other provinces (Ducatez et al., 2008; Eltahir et al., 2011; Zhang et al., 2013; Li et al., 2017). Since 2014, CAV outbreaks have occurred frequently in southern China, particularly in the Guangdong province (Zeng et al., 2021). According to a study conducted on the live poultry market in southern China, the virus was present in up to 87% of the birds (Ducatez et al., 2008). Recent surveys of chicken farms have revealed that the CAV seropositivity rate is high in three provinces of China, e.g., Zhejiang, Jiangsu, and Anhui (Zeng et al., 2021). It is becoming difficult to control the virus spread because of its great genetic diversity depending upon region. Recently, another novel *Gyrovirus* has been identified in the feces of humans that have genomic similarities to CAV (Rao et al., 2022). Therefore, to better understand the genetic evolution of CAV, we evaluated the complete genomes of the globally isolated CAVs between 1991–2020 by analyzing the phylogenetic, phylogeographic, and recombination characteristics of the virus.

## Materials and methods

### Dataset

In this study, we accessed the NCBI GenBank database and retrieved all the full-length genome sequences of CAV isolated globally from 1991 to 2020 (a total of 243), including 170 from China, 24 from Egypt, 22 from Turkey, 7 from Malaysia, 3 from Vietnam, Germany, USA, and Brazil respectively, 2 from Argentina and Australia respectively, and 1 from South Korea, India, Iran, and Japan, respectively. The virus strains were identified using their GenBank ID, name, collection year and country/region [GenBank ID: virus/strain-collection year-country/region].

### Phylogenetic tree construction and genomic similarity analysis

All the 243 full-length nucleotide sequences of CAV were aligned with the ClustalW using the MEGA11 software (Tamura et al., 2021) and edited using the BioEdit v7.2.5 package (Hall, 1999). Following the alignment, the ML (maximum likelihood) phylogenetic tree was inferred with the best-fitting model TIM3+F+I+G4 using the IQ-TREE v1.6.12 (Trifinopoulos et al., 2016). The tree was modified and visualized with the help of FigTree v1.4.^1^ In addition, the genetic similarity map of selected representative sequences was achieved using SimPlot v3.5.1 (Lole et al., 1999).

### Phylogeographic network of full-length chicken anemia virus genomes, 1991–2020

The phylogeographic network depicts the regional level spread, portrays the genetic linkages between the intra-specific sequences, and bridges the population genetic data by inferring their relationships (Leigh and Bryant, 2015). Thus, all the CAV full-length genomic sequences were modified and exported into Nexus format using the MEGA11 software (Tamura et al., 2021). The phylogeographic network was mapped by inferring the Minimum Spanning Network (MSN) implemented by the PopArt v1.7 (Leigh and Bryant, 2015). The network included fifteen groups from fourteen different countries, e.g., Mainland China (125 sequences), Taiwan region of China (45), Egypt (24), Turkey (22), Malaysia (7), USA (3), Brazil (3), Vietnam (3), Germany (3), Argentina (2), South Korea (1), India (1), Iran (1), Japan (1), and Australia (2).

### Recombination analysis of full-length chicken anemia virus genomes, 1991–2020

The recombination events among the 243 complete genome sequences of CAV were assessed using the RDP4 software package (Martin et al., 2015). The potential recombination events were detected using each of the seven algorithms implemented by the RDP4 software, e.g., RDP, GENECONV, SiScan, 3seq, Bootscan, Chimaera, and MaxChi. The recombination events were accepted as real when confirmed by at least three of these seven methods.

### Animo acids variability analysis of chicken anemia virus

The complete nucleotide sequences of all the 243 CAV ORFs (ORF1, ORF2, and ORF3, encoding the VP1, VP2, and VP3 proteins, respectively) were separately retrieved from the NCBI database and were aligned and translated into amino acids sequences using the MEGA11 software (Tamura et al., 2004; Kumar et al., 2018). The amino acids variability landscape was achieved with the Wu-Kabat variability coefficient method implemented by the PVS (protein variability server; Garcia-Boronat et al., 2008). The variability coefficient is calculated using the following formula: variability = N*k/*n*, where *N* is the number of sequences in the alignment, k is the number of different amino acids at a given position, and *n* represents the time that the most commonly recognized amino acid at that position is available.

## Results

### Genotyping full-length chicken anemia virus genomes

A total of 243 complete genome sequences of CAV, isolated from 1991 to 2020, were analyzed to determine the phylogenetic, phylogeographic, and recombination patterns of CAVs and track the global spread of CAVs. All the full-length genomic sequences were aligned, and an ML (maximum likelihood) phylogenetic tree was constructed based on 1,000 bootstraps using best-fitting model TIM3+F+I+G4 in the IQ-TREE v1.6.12 (Trifinopoulos et al., 2016).

**Figure 1:**
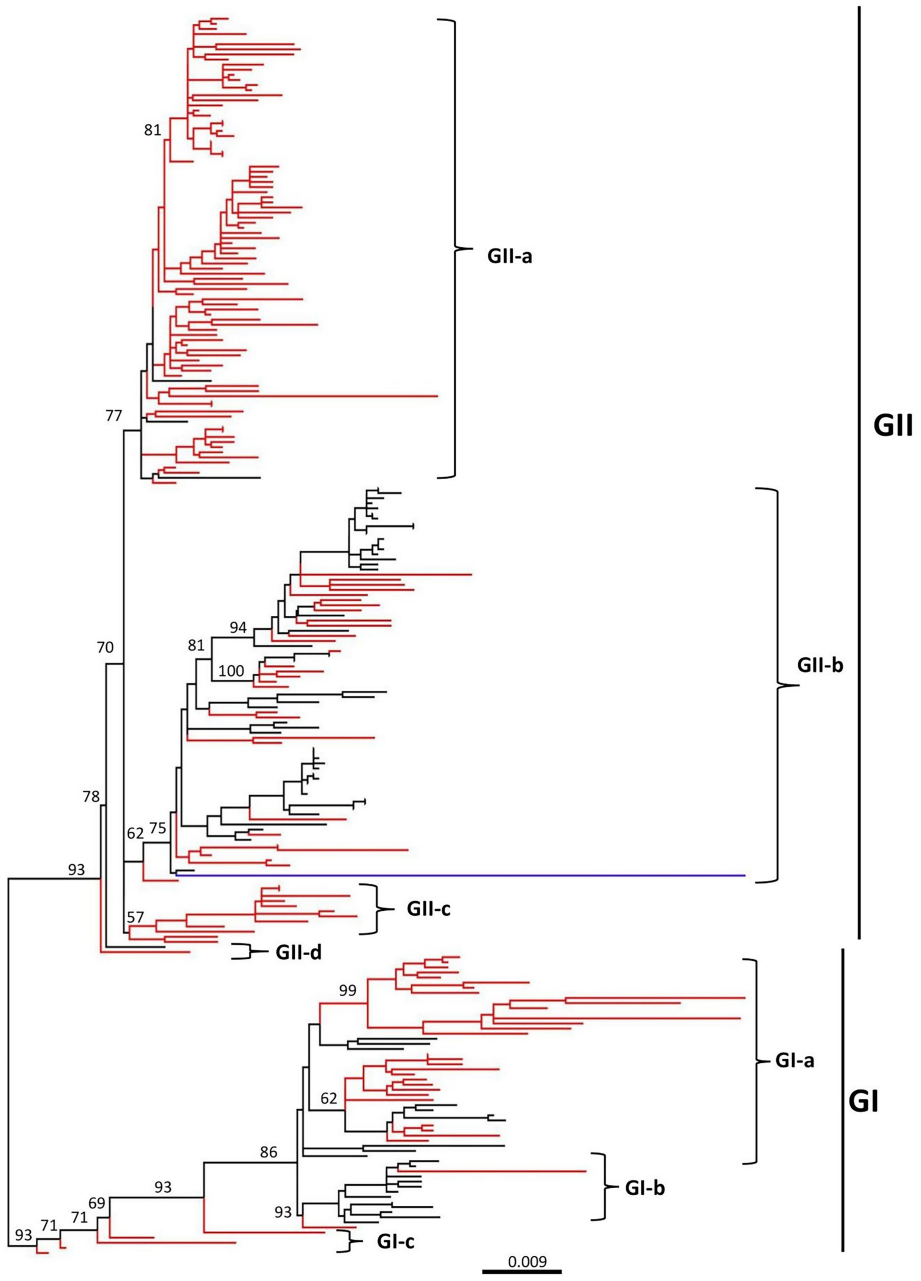
Phylogenetic tree based on the full-length genome sequences of CAV strains, 1991–2020. ML (maximum likelihood) phylogenetic tree of 243 full-length genome sequences of CAV classified all strains into two major clades, GI and GII. GI can be further classified into three sub-clades (GI-a, GI-b, and GI-c), while GII into four sub-clades (GII-a, GII-b, GII-c, and GII-d). The major clades and sub-clades of the CAV are indicated. The percentage of replicate trees in which the associated taxa clustered together in the bootstrap test (1,000 replicates) are indicated at each node. The evolutionary distances were computed using the best-fit substitution model TIM3+F+I+G4. The tree was visualized and modified to be proportional using FigTree v1.4. The branches in red color represents the CAVs isolated in China, while the blue color branch represents the CAV vaccine strain (GenBank ID: EF683159.1). The detailed information about the viruses in the tree can be seen in Supplementary Figure S1.

The phylogenetic tree indicated that all the CAV strains are grouped into two distinct clades GI and GII, where the GI clade is further divided into three sub-clades (GI-a, GI-b, and GI-c), while the GII into four sub-clades (GII-a, GII-b, GII-c, and GII-d) (Figure 1; Supplementary Figure S1). The strains from China (125 strains from China mainland and 45 from Taiwan) were the most dominant and were present in all sub-clades. The GI-a consisted of strains from China (33), Egypt (6) and the USA (1), while the GI-b was the most diverse sub-clade consisting of strains from China (2), Japan (1), Vietnam (1), Malaysia (2), Egypt (2), Brazil (3), Argentina (2), and Australia (1). In addition, five of the strains isolated in China mainland and Taiwan region, e.g., GX1904B (GenBank ID: MN103406.1), CIAV-Dog (GenBank ID: KU645524.1), SD24 (GenBank ID: AY999018.1), SD22 (GenBank ID: DQ141673.1), and 1840TW (GenBank ID: MT799766.1), clustered separately within the GI clade (shown as GI-c) and were more distanced from all the remaining strains (Figure 1; Table 1; Supplementary Figure S1).

**Table 1:** Geographic distribution of CAV full-length genomes-based genotypes, 1991–2020.

Genotypes	Asia	Africa	Europe	America	Oceania	Total
GI-a	China (33)	Egypt (6)	–	USA (1)	–	40
GI-b	China (2), Japan (1), Vietnam (1), Malaysia (2)	Egypt (2)	Argentina (2)	Brazil (3)	Australia (1)	14
GI-c	China (5)	–	–	–	–	5
GII-a	China (87), Turkey (3), South Korea (1), Vietnam (1)	–	–	–	–	92
GII-b	China (30), Turkey (19), Vietnam (1), Malaysia (4), India (1), Iran (1)	Egypt (16)	Germany (3)	USA (2)	Australia (1)	78
GII-c	China (12)	–	–	–	–	12
GII-d	China (1), Malaysia (1)	–	–	–	–	2
Total	206	24	5	6	2	243

On the other hand, the GII clade clustered the highest number of strains, where the GII-a encompasses only strains from Asia, e.g., China (87), Turkey (3), South Korea (1), and Vietnam (1). GII-b was the most diverse sub-clade within the GII, clustering strains from China (30), Turkey (19), Vietnam (1), Malaysia (4), India (1), Iran (1), Egypt (16), USA (2), Australia (1), and Germany (3), while the GII-c sub-clade was limited to strains only from China (12). Similar to the GI clade, two of the strains isolated in China and Malaysia, respectively, clustered separately from all the remaining strains within the GII clade, including the SD1505 isolated in China in 2015 (GenBank ID: KU645523.1) and the LF4 isolated in Malaysia in 2005 (GenBank ID: AY839944.2) (Figure 1; Table 1; Supplementary Figure S1). Interestingly, the CAV vaccine strain reported in Australia in 2007 (GenBank ID: EF683159.1) clustered within the GII-b and was genetically closer to the D02152 isolated in United States in 2003 (GenBank ID: AF311892.2). Similarly, the SDAUC-VacChina strain (GenBank ID: MF614011.1), which is reported to be a Newcastle disease virus-attenuated vaccine co-contaminated with fowl adenovirus and chicken infectious anemia virus (Su et al., 2018), clustered within the GI-a sub-clade (Figure 1; Supplementary Figure S1).

**Figure 2:**
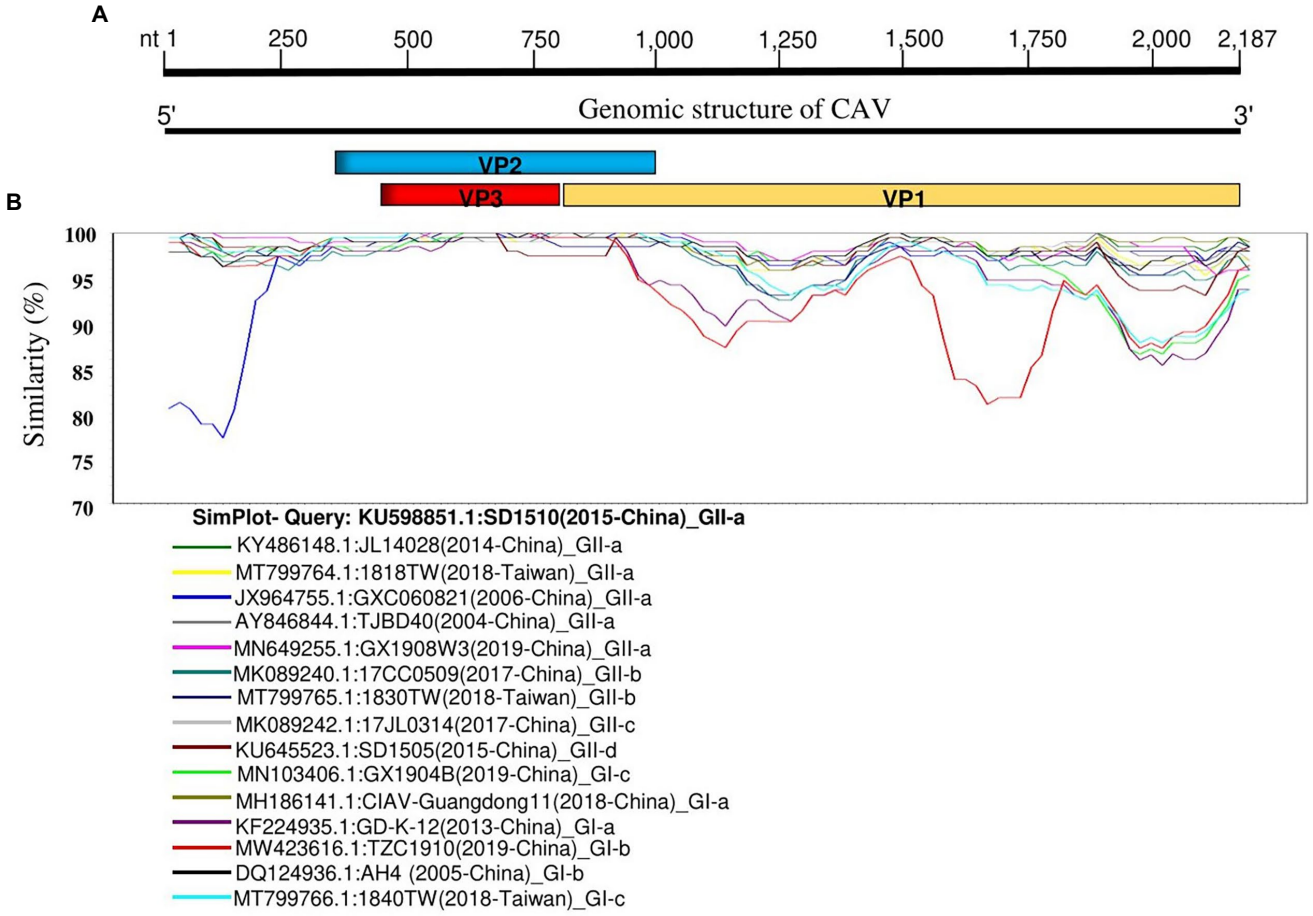
Genetic similarity map of the full-length genome sequences of representative CAV strains. **(A)** Schematic diagram of CAV complete genome structure. From 5′ end to 3′ end are the three overlapping ORFs that encode for the VP2, VP3, and VP1 proteins. **(B)** SimPlot similarity analysis results using the SD1510 (2015-China) strains within the GII-a (GenBank ID: KU598851.1) as the query sequence to compare with the fifteen other representative strains from each subclade.

Since the CAV isolates showed great diversity, we further analyzed the genetic similarity of CAV full-length genomes using fifteen representative strains from each sub-clade and SD1510 strain (2015-China) (GenBank ID: KU598851.1) as the query. The genetic similarity analysis indicated a great diversity, corroborating our phylogenetic tree. The nucleotide position encoding the VP1 protein (nt1000-2,187) showed the lowest similarity level (<95%) and indicated two distinct groups (GI and GII). The nucleotide position around 1–1,000 (encoding VP2 and VP3 proteins) is shown to be the most conserved region. Moreover, the GXC060821 strain (GenBank ID: JX964755.1, 2006-China) in GII-a and the TZC1910 (GenBank ID: MW423616.1, 2019-China) in GI-b showed the lowest similarity (<85%) at nt positions 1–250 and 1,500–1,800, respectively, (Figure 2).

### Phylogeographic network of full-length chicken anemia virus genome sequences

**Figure 3:**
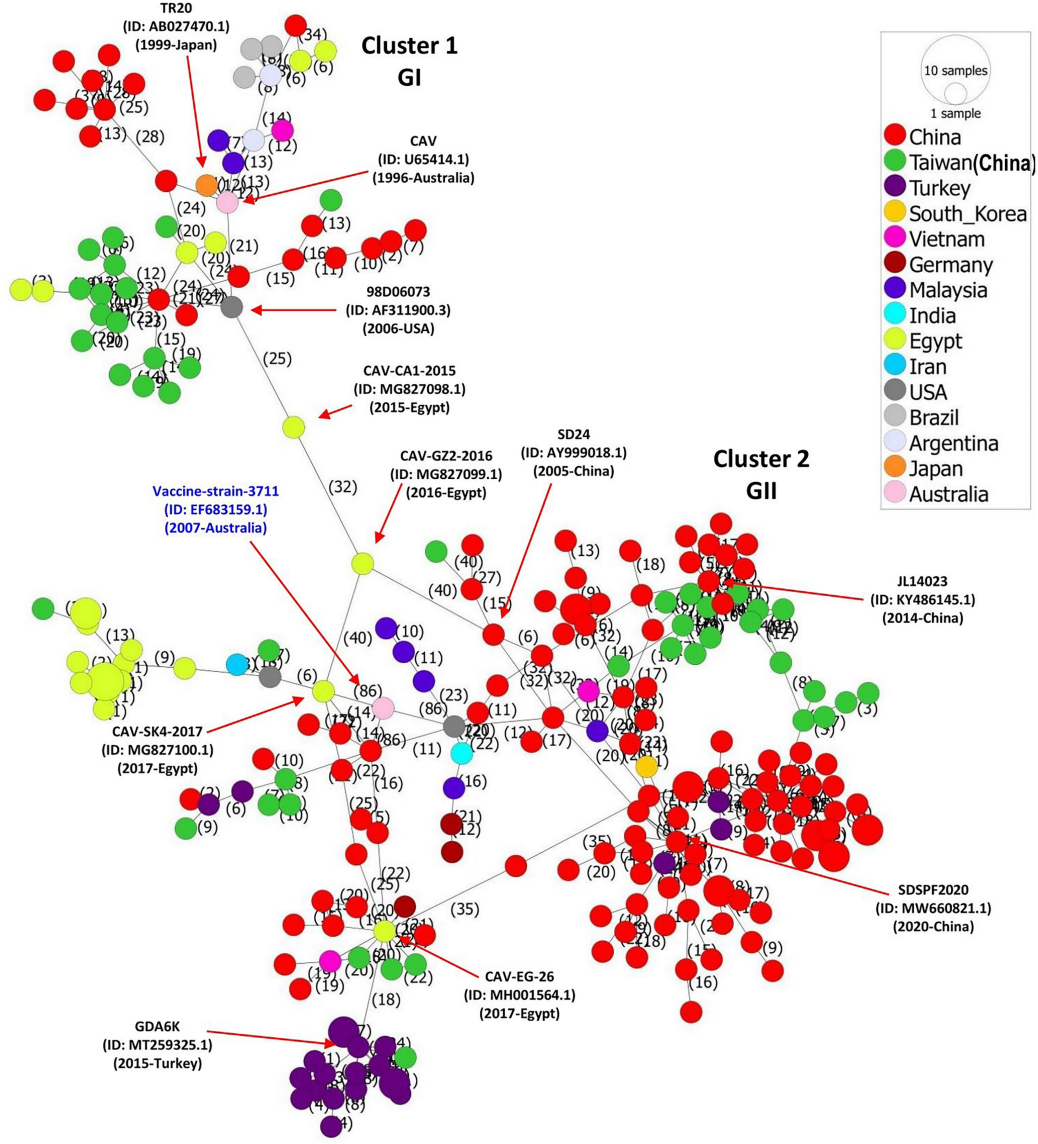
Phylogeographic network analysis of the full-length genome sequences of CAV, 1991–2020. The phylogenetic network of 243 full-length genomes of CAVs was inferred using the MSN network implemented by PopArt v1.7. Cluster 1 and Cluster 2 (relevant to GI and GII clades, respectively) are connected by three strains isolated in USA and Egypt, e.g., 98D06073 (GenBank ID: AF311900.3,2006-USA), CAV-CA1-2015 (GenBank ID: MG827098.1, 2015-Egypt), and CAV-GZ2-2016 (GenBank ID: MG827099.1,2016-Egypt) respectively, following 25 and 32 mutational substitutions within these strains. The number represents the mutational steps and substitutions. Each color represents a different country or region.

We constructed the CAV full-length sequences-based phylogeographic network to further evaluate the regional level spread and potential mutational steps of CAVs. In consistence with our phylogenetic and genetic similarity results, the phylogeographic analysis indicated a huge diversity of CAVs. The network analysis showed two major clusters (relative to GI and GII clades) with multiple further sub-branches within each cluster, where the strains isolated in China mainland and Taiwan region dominated the network (Figure 3). Interestingly, Cluster 1 and Cluster 2 are connected by three strains isolated in the USA and Egypt, e.g., 98D06073 (GenBank ID: AF311900.3, 2006-USA), CAV-CA1-2015 (GenBank ID: MG827098.1, 2015-Egypt) and CAV-GZ2-2016 (GenBank ID: MG827099.1, 2016-Egypt), respectively, following 25 and 32 mutational substitutions within these strains. Similarly, the strains isolated in Brazil and Argentina are connected to the early isolated strain (GenBank ID: U65414.1, 1996-Australia) and TR20 (GenBank ID: AB027470.1, 1999-Japan) through a short mutational branch, while the majority of strains isolated in Turkey are connected to the CAV-EG-26 strain (GenBank ID: MH001564.1, 2017-Egypt) (Figure 3). In addition, the CAV vaccine strain 3,711 (GenBank ID: EF683159.1, 2007-Australia) is connected to the 98D02152 (GenBank ID: AF311892.2, 2003-USA) and CAV-SK4-2017 (GenBank ID: MG827100.1,2017-Egypt), both of which were clustered within the GII-b in the phylogenetic tree (Figure 1; Supplementary Figure S1). These results speculate the spread of CAVs from Japan to China to Egypt and subsequently to multiple regions of the globe. The spread of CAVs between different regions may have occurred through the trade of birds and their products or by natural carriers.

### Recombination analysis

**Figure 4:**
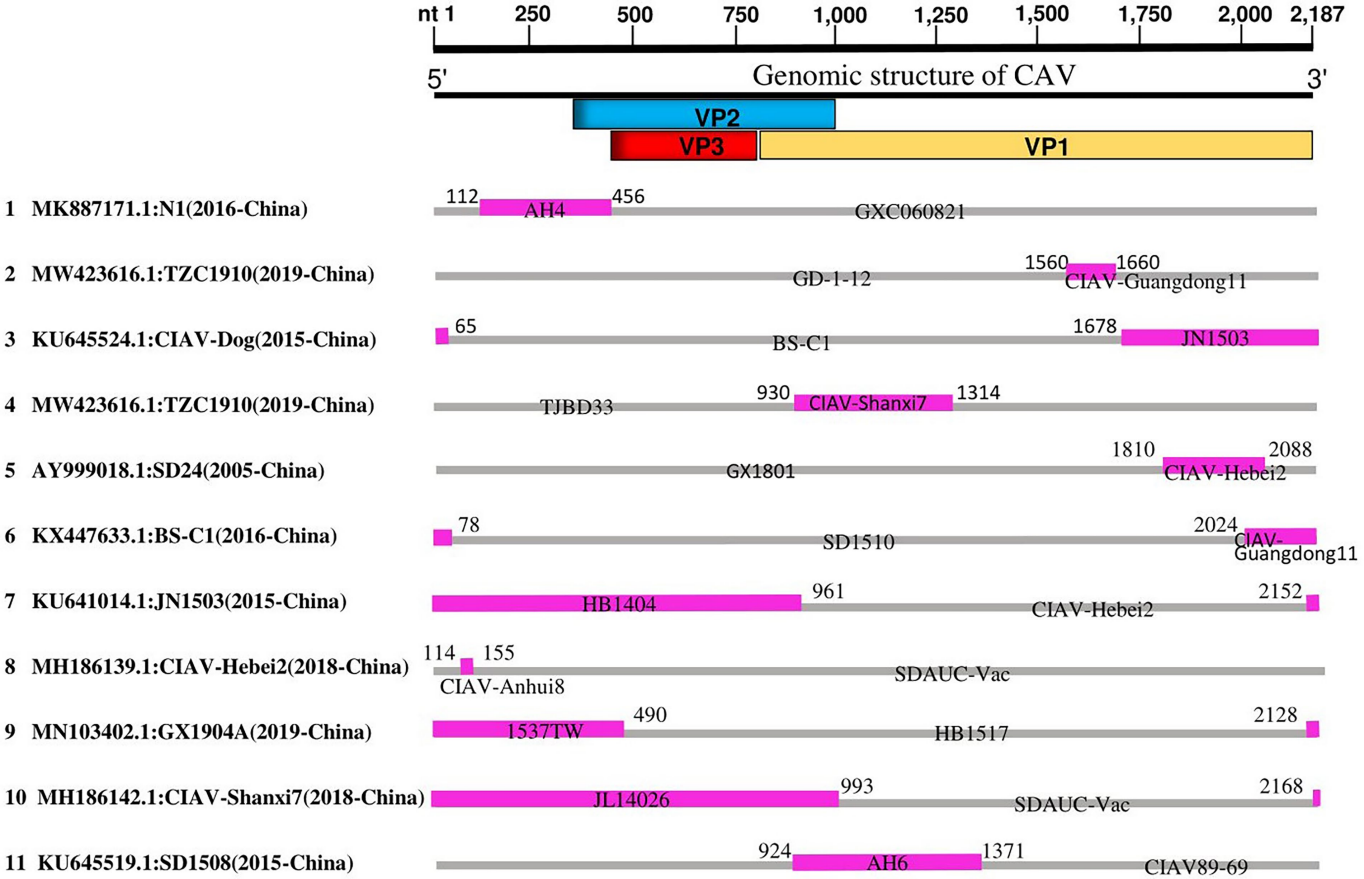
Genetic recombination analysis of 243 full-length genomes of CAV, 1991–2020. Diagram on the top showing the full-length genome of CAV and the corresponding regions encoding the VP1, VP2, and VP3 proteins. The numbers indicate the nucleotide positions relative to the genome of CAV. Schematic representation of the eleven potential recombination events listed in Table 2. The serial number of the recombination events and the description of potential recombinants (GenBank ID: virus name/collection year-country/region) are shown on the left. The pink and gray blocks represent the DNA regions from minor and major parent viruses, respectively. The numbers on the top of filled pink blocks indicate the nucleotide positions of breakpoints relative to the genome sequences of the corresponding recombinant viruses on the left.

Since the phylogenetic, genetic similarity, and phylogeographic network analyses revealed possible mutations and genetic exchanges, we evaluated the occurrence of recombination among the 243 full-length genome sequences of CAVs. The RDP4 software package (Martin et al., 2015) was used to map the recombination patterns and genomic breakpoints. We identified a total of eleven recombination events, among which, ten events were inter-genotype (Events 1–10), and only one was intra-genotype (Event 11; Table 2). As shown in Figure 4, four events (Events 2, 3, 5 and 6) occurred within the VP1 protein coding region (breakpoints beginning at nt 1,560, 1,678, 1,810, 2,024, and ending at nt 1,660, 65, 2,088, and 78, respectively). Similarly, two events (Event 4 and 11) occurred within the VP1/VP2 proteins encoding regions (breakpoints beginning at nt 930, 924 and ending at nt 1,314, 1,371 respectively), one event (Event 1) within VP2 (beginning at nt 112 and ending at nt 456), one (Event 8) within the 5′ end region (beginning at nt 114 and ending at nt 155), while three events (Event 7, 9, and 10) encompassed all the three protein VP1/VP2/VP3 coding regions (beginning at nt 2,152, 2,128, 2,168, and ending at nt 961, 490, 993, respectively; Figure 4).

**Table 2:** Identification of 11 potential recombination events in the genome of CAVs isolated during 1991–2020.

Event serial NO.	Recombinant		Minor parent		Major parent		Detection methods
	GenBank ID: Virus name (Year-Country/region)	Genogroup	GenBank ID: Virus name (Year-Country/region)	Genogroup	GenBank ID: Virus name (Year-Country/region)	Genogroup	R,G,B,M,C,S,T
1	MK887171.1:N1(2016-China)	GII-a	DQ124936.1:AH4(2005-China)	GI-b	JX964755.1:GXC060821(2006-China)	GII-a	**+++++++**
2	MW423616.1:TZC1910(2019-China)	GI-b	MH186141.1:CIAV-Guangdong11(2018-China)	GI-a	JX260426.1:GD-1-12 (2012-China)	GII-a	**++−++++**
3	KU645524.1:CIAV-Dog(2015-China)	GI-c	KU641014.1:JN1503(2015-China)	GI-a	KX447633.1:BS-C1(2016-China)	GII-c	**+++++++**
4	MW423616.1:TZC1910(2019-China)	GI-b	MH186142.1:CIAV-Shanxi7(2018-China)	GI-a	AY843527.2:TJBD33(2005-China)	GII-b	**+−+++++**
5	AY999018.1:SD24(2005-China)	GI-c	MH186139.1:CIAV-Hebei2(2018-China)	GI-a	MK484614.1:GX1801(2018-China)	GII-a	**+++++++**
6	*KX447633.1:BS-C1(2016-China)	GII-c	MH186141.1:CIAV-Guangdong11(2018-China)	GI-a	KU598851.1:SD1510(2015-China)	GII-a	**+++−−−+**
7	*KU641014.1:JN1503(2015-China)	GI-a	KU645514.1:HB1404(2014-China)	GII-a	MH186139.1:CIAV-Hebei2(2018-China)	GI-a	**+++++++**
8	*MH186139.1:CIAV-Hebei2(2018-China)	GI-a	MH186140.1:CIAV-Anhui8(2018-China)	GII-b	MF614011.1:SDAUC-Vac(2017-China)	GI-a	**++−−−−+**
9	MN103402.1:GX1904A(2019-China)	GI-a	MT795930.1:1537TW(2016-Taiwan, China)	GII-b	KU645516.1:HB1517(2015-China)	GII-b	**++−−+−+**
10	*MH186142.1:CIAV-Shanxi7(2018-China)	GI-a	KY486147.1:JL14026(2014-China)	GII-a	MF614011.1:SDAUC-Vac(2017-China)	GI-a	**+−+++++**
11	KU645519.1:SD1508(2015-China)	GII-a	DQ124935.1:AH6(2005-China)	GII-b	JF507715.1:CIAV89-69 (1991-South_Korea)	GII-a	**+−−−−++**

R, RDP; G, GENECONV; B, BootScan; M, MaxChi; C, Chimaera; S, SiScan; T, 3seq. +, verified; −, not verified. *The major or minor parent may be the actual recombinant due to the possibility of misidentification.

The results showed that the CAV strains isolated in China are highly active and appeared in all recombination events. The unique intra-genotype recombination event (Event 11) involved a strain from South Korea, CIAV89-69 (GenBank ID: JF507715.1, 1991-South Korea), as a major parent. Importantly, two of the strains that clustered separately in our phylogenetic tree within the GI-c, e.g., CIAV-Dog (GenBank ID: KU645524.1, 2015-China) and SD24 (GenBank ID: AY999018.1, 2005-China), were found to be recombinants (Events 3 and 5, respectively), the AH4 strain (GenBank ID: DQ124936.1, 2005-China) that clustered as a distanced strain within the GI-b appeared as a minor parent of the recombinant N1 of Event 1 (GenBank ID: MK887171.1, 2016-China) (Figure 4; Table 2); meanwhile, the strain TZC1910 (MW423616, 2019-China) that showed the lowest similarity level (<85% at VP1 gene) is identified as a recombinant event (Event 2) with beginning and ending breakpoints located within VP1 coding region (nt1560 and nt1660, respectively).

To further validate the evidence of the identified recombination events, we constructed three separate phylogenetic trees based on the three fragments of the CAV genome. The first fragment (nt 1–450) corresponds to the 5′ end to the beginning of VP1 and VP2 ORFs, the second fragment (nt 1–900) encodes for the VP1 and VP2, and the third fragment (nt 1,800-2,187) is relative to the 3′ end of the VP1 ORF. The short fragments-based phylogenetic trees are not superimposable on each other (Supplementary Figures S2A–C). For instance, the recombinant in event 1 nested with its minor parent in the first tree (Supplementary Figure S2A) but with its major parents in the second (Supplementary Figure S2B) and third (Supplementary Figure S2C) trees. The results indicate that the recombination of CAV genomes drives the rise of new virus lineages.

### Amino acid variability pattern of chicken anemia virus proteins

**Figure 5:**
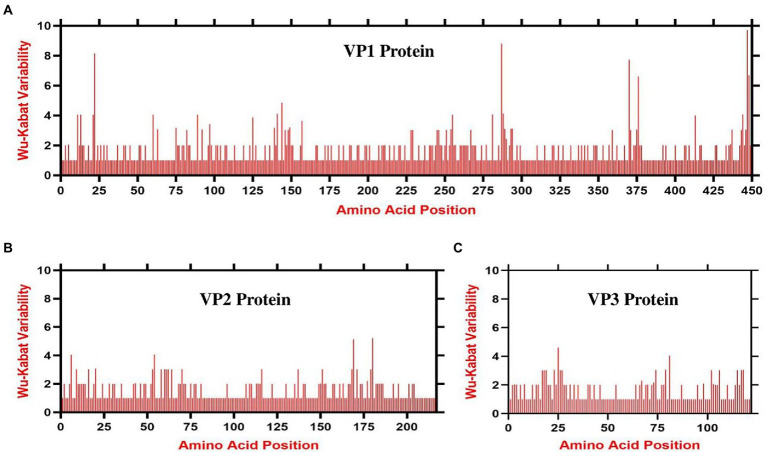
Amino acid variability landscape of full-length proteins of CAV, 1991–2020. The plot represents amino acid variations in **(A)** ORF1-encoded VP1 capsid protein, **(B)** ORF2-encoded VP2 protein and **(C)** ORF3-encoded VP3 apoptin protein. The ORFs nucleotide sequences were used to acquire their consensus amino acids sequence using the Wu-Kabat variability coefficient implemented by PVS. Y-axes represent the Wu-Kabat variability coefficient values, where the estimation limit is 1. Above the limit of 1 represents variations. X-axes represent the amino acid positions.

The amino acid variability patterns across the three VP1, VP2, and VP3 proteins of CAV were assessed using the Wu-Kabat variability method offered by the PVS. The consensus sequence of the VP1 protein consisted of 449 amino acids, the VP2 of 216 amino acids, and the VP3 protein contained 121 amino acids. The Wu-Kabat variability coefficient indicated significant variability across all three proteins, where the values in multiple regions exceeded the estimation limit of 1 (Figures 5A–C). The VP1 protein was indicated to be the most variable, especially at the aa region 11–30, 285–294, and 370–378 (highest values 8, 8, and 7, respectively; Figure 5A). Similarly, the VP2 aa position 149–186 and VP3 aa position 2–35 indicated great variability (highest values 5 and 4 respectively) (Figures 5B,C). These results suggest that the amino acids across all three proteins varied greatly during 1991–2020.

## Discussion

The chicken anemia virus is responsible for immunosuppressive poultry disease CIA (Pope, 1991; Schat, 2009; Liu et al., 2022), causing huge economic losses to the poultry industry in many parts of the world (Schat, 2009; Fatoba and Adeleke, 2019). According to the International Committee on Taxonomy of Viruses (ICTV), the virus is placed in the family *Anelloviridae* and genus *Gyrovirus* (Rosario et al., 2017; ICTV, 2020; Di Francesco et al., 2022), however, there is no classification on the clades and sub-clades level as per ICTV, and CAV strains are usually classified into different groups and sub-groups by the researchers reporting the new strains (Craig et al., 2009; Zhang et al., 2013; Liu et al., 2022; Zhang et al., 2022). Previously, CAVs were sorted into five groups (A to E) based on 54 partial genomic sequences (Zhang et al., 2013), or into four major groups (A, B, C, and D) based on 55 VP1, VP2, and VP3 complete coding sequences (Eltahir et al., 2011). Similarly, 121 complete genomes of CAVs were divided into eight lineages (Li et al., 2017). Herein, we accessed the NCBI database and retrieved the available full-length genome sequences (a total of 243) isolated globally from 1991 to 2020 and grouped them into two distinct clades, GI and GII, where the GI clade is further divided into three sub-clades (GI-a, GI-b, and GI-c), while GII into four sub-clades (GII-a, GII-b, GII-c, and GII-d). The differences in CAV classification proposed by previous studies are related to different classification methods and inclusion criteria of the viruses, where researchers have analyzed different reference strains with different sequence lengths. For example, 54 partial genomes-based and 55 complete genome-based studies generated the CAV phylogenetic trees using the Neighbor-Joining approach with MEGA software (Eltahir et al., 2011; Zhang et al., 2013), while the 121 complete genomes-based study analyzed the CAV genetic evolution using the ML (maximum likelihood) method with RAxML software (Li et al., 2017). We analyzed the available full-length genome sequences and inferred the ML phylogenetic tree with the best-fitting model using the IQ-TREE multicore version 1.6.12 (Trifinopoulos et al., 2016). Our results provide the latest and most robust phylogenetic analysis that may be used to place the existing and newly reported strains.

Since the first case of CAV in 1979 in Japan (Yuasa et al., 1979), the virus has spread to most of the poultry-breeding countries (Rosenberger and Cloud, 1989; Craig et al., 2009; Abdel-Mawgod et al., 2018; Quaglia et al., 2021; Techera et al., 2021). Thus, we analyzed the phylogeographic network of all CAV strains available to date. The phylogeographic analysis also revealed two major clusters corresponding to the GI and GII clades of the phylogenetic analysis. CAV outbreaks have been reported to be frequently occurred in China since 2014, especially in the southern region of the country (Zeng et al., 2021). Our results speculate the spread of the virus from Japan to China, China to Egypt and other countries. A previous study has also reported similar results, which reported that the CAV spread from Japan to Africa and South America during 1984–1985 and from China to Egypt during 1984 (Techera et al., 2021). These results are also in agreement with the historical records, showing the evidence that during the 1980s, China and Japan were the two main producers and exporters of poultry, which may have facilitated the CAV introduction into other parts of the world (FAOSTAT, 2018).

Genetic recombination is crucial in the evolution of viruses and plays a significant role in maintaining or generating diversity in viruses. CAVs exhibit a low efficiency of recombination as they are DNA viruses. Nevertheless, earlier studies have provided evidence of genetic recombination events in CAVs (Eltahir et al., 2011; Tan et al., 2020; Li et al., 2021; Liu et al., 2022). In this study, we detected eleven recombination events among the CAVs isolated from 1991–2020. The published studies suggest that recombination within CAVs could occur across the coding and non-coding regions (Li et al., 2017; Tan et al., 2020), which is consistent with the findings in our study.

The CAV genome contains three overlapping ORFs encoding the VP1, VP2, and VP3 proteins (Rosenberger and Cloud, 1998; Lacorte et al., 2007). The amino acid variability analysis in this study indicated that the VP1 protein had the highest variability, concentrated in some hypervariable regions, e.g., regions aa 11–30, aa 285–294, and aa 370–378. This finding is consistent with a recent report, which shows that the VP1 protein has the highest mutation rate, and sites of amino acid variations are concentrated in hypervariable regions (Liu et al., 2022). These concentrated variation sites within the VP1 seem to be related to the replication and pathogenicity of the virus (Renshaw et al., 1996; Yamaguchi et al., 2001; Todd et al., 2002). In contrast to the notion that VP2 and VP3 are the most conserved proteins with no universal mutations (Liu et al., 2022), our results indicate several variable regions within VP2 and VP3 proteins that exceeded the estimation limit. Though, the binding site of CAV in chickens remains to be elucidated, VP1 and VP2 proteins are the prime targets in designing vaccines to induce neutralizing antibodies (Moeini et al., 2011a), and our findings may provide valued information for vaccine design as well as better understanding of CAV pathogenesis. Our findings indicate that the amino acids across all three proteins have greatly varied during 1991–2020. This divergence is also clearly evidenced from the existence of various sub-clades within each clade in the phylogenetic tree (Figure 1) and multiple mutational branches within the phylogeographic network of the CAVs (Figure 3). Therefore, we speculate that substantial genetic mutation and recombination in CAV genomes were involved in generation of new viral lineages.

In summary, this study provides the latest insights into the phylogenetic characteristics, geographic distribution and genetic variability patterns of the chicken anemia virus based on the full-length genomic sequences isolated in 1991–2020. The classification of CAVs into two major clades with further sub-clades may offer a robust system of placing the existing and future strains. In addition, genetic recombination and amino acid variability indications may be used to determine the pathogenicity and design effective vaccines to facilitate the prevention and control measures of CAVs.

## Data availability statement

The original contributions presented in the study are included in the article/Supplementary material, further inquiries can be directed to the corresponding author.

## Ethical statement

For this retrospective type of study, formal consent is not required. Statement on the welfare of animals is not applicable as sample collection from animals has been done before.

## Author contributions

PS and LX: conceptualization. PS, AB, XC, and SS: data analysis. PS: visualization and writing. CW and LX: administration. PS, AB, and LX: manuscript revision. All authors contributed to the article and approved the submitted version.

## Funding

The Program of Introducing Talents of Discipline to Universities (D21004).

## Conflict of interest

The authors declare that the research was conducted in the absence of any commercial or financial relationships that could be construed as a potential conflict of interest.

## Publisher’s note

All claims expressed in this article are solely those of the authors and do not necessarily represent those of their affiliated organizations, or those of the publisher, the editors and the reviewers. Any product that may be evaluated in this article, or claim that may be made by its manufacturer, is not guaranteed or endorsed by the publisher.

## Footnotes

^1^ http://tree.bio.ed.ac.uk/software/figtree/
