# Supplementary figures and images for: Genetic diversity, distribution, and evolution of chicken anemia virus: A comparative genomic and phylogenetic analysis
Source: Front Microbiol. 2023 Mar 9;14:1145225. doi: 10.3389/fmicb.2023.1145225 (PMC10034120; doi:10.3389/fmicb.2023.1145225)

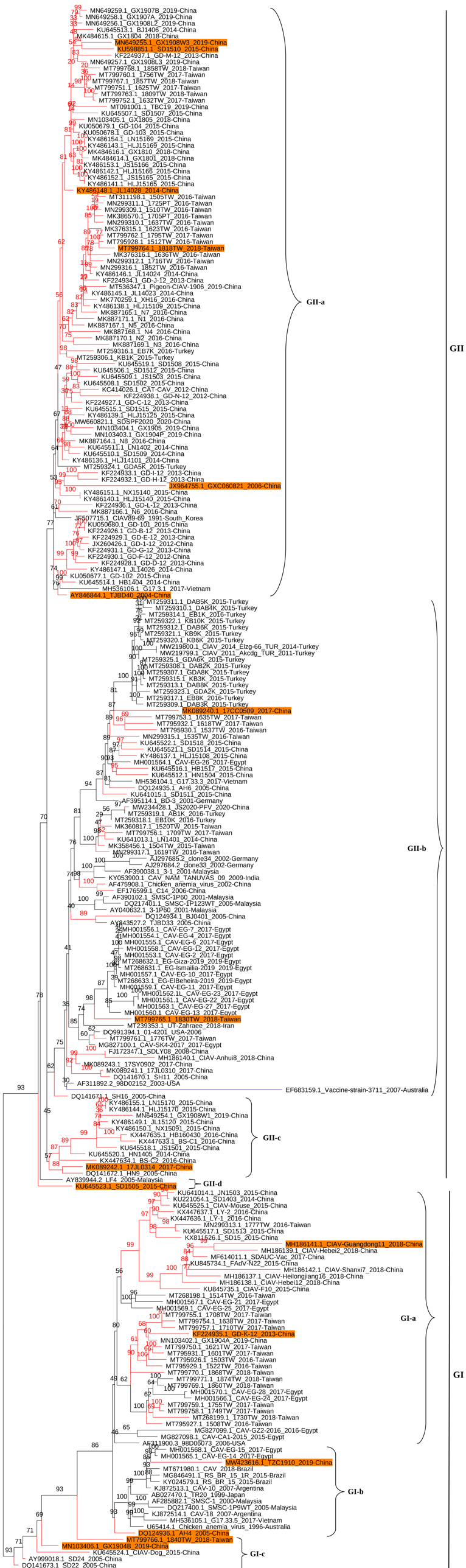

Supplement: Supplementary file 1 [file Data_Sheet_1.PDF]
