# Supplementary material for: Genetic diversity, distribution, and evolution of chicken anemia virus: A comparative genomic and phylogenetic analysis
Source: Front Microbiol. 2023 Mar 9;14:1145225. doi: 10.3389/fmicb.2023.1145225 (PMC10034120; doi:10.3389/fmicb.2023.1145225)

Supplementary Figure 2. The phylogenetic analysis based on the indicated genomic fragments of CAVs involved in recombination events. Phylogenetic trees based on **(A)** region nt 1-450, **(B)** region nt 1-900 and **(C)** region 1800-2187 of CAV genomes. Viruses involved in recombination events 1, 3, 4, 5, 9 and 11 were labeled with different symbols in the phylogenetic trees constructed using the Neighbor-Joining method and 1000 bootstraps. The recombinant, major, and minor parents were indicated with red, yellow, and blue colors, respectively. The nucleotide sequence is relative to the China strain TZC1910 (GenBank ID: MW423616.1).

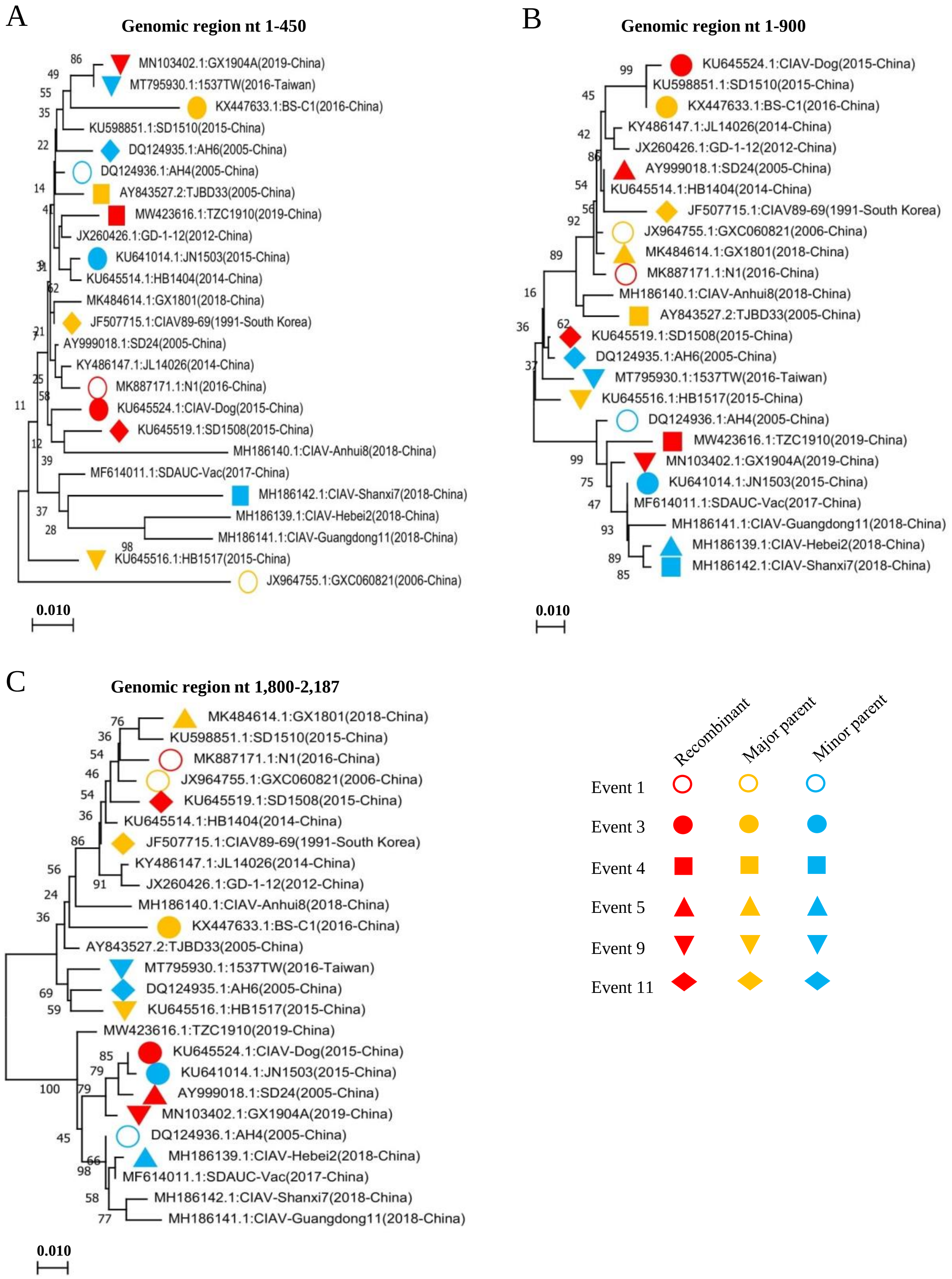

Supplement: Supplementary file 2 [file Data_Sheet_2.PDF]
